# The Effects of Vasoactive Medications on Mean Circulatory Filling Pressure, Venous Resistance, Systemic Vascular Resistance, Cardiac Index, and Oxygen Extraction After Pediatric Heart Transplant: Leveraging High-Fidelity Physiologic Data

**DOI:** 10.3390/children13020262

**Published:** 2026-02-13

**Authors:** Julia Behrend, George Hoffman, John N. Kheir, Wesam Sourour, Anna Joong, Rohit S. Loomba

**Affiliations:** 1 Ann & Robert H. Lurie Children’s Hospital, Chicago, IL 60611, USA; jbehrend@luriechildrens.org (J.B.); wsourour@luriechildrens.org (W.S.); ajoong@luriechildrens.org (A.J.); 2 Feinberg School of Medicine, Northwestern University, Chicago, IL 60611, USA; 3 Children’s Wisconsin, Wauwatosa, WI 53226, USA; ghoffman@mcw.edu; 4 Department of Pediatrics, Medical College of Wisconsin, Wauwatosa, WI 53226, USA; 5 Department of Cardiology, Boston Children’s Hospital, Boston, MA 02115, USA; john.kheir@cardio.chboston.org; 6 Department of Pediatrics, Harvard Medical School, Boston, MA 02115, USA

**Keywords:** vasoactive, inotrope, mean circulatory filling pressure, venous resistance, venous return, epinephrine, milrinone, dopamine, vasodilator, venodilator

## Abstract

**Highlights:**

**What are the main findings?**

- High-fidelity physiologic data can help estimate venous parameters such as mean circulatory filling pressure, venous resistance, and venous return.
- Vasoactive medications have different effects on the arterial and venous systems.

**What is the implication of the main finding?**

- Venous parameters can be estimated at the bedside and can help clarify the physiology.
- Venous parameters can help select appropriate vasoactive medications.

**Abstract:**

*Background*: The physiologic effects of vasoactive medications on the venous circulation remain incompletely understood. Contemporary bedside management often emphasizes the arterial circulation, whereas Guytonian physiology emphasizes the venous circulation and mean circulatory filling pressure in determining steady-state cardiac output. The primary aim of this study was to characterize the effect of vasoactive medications on mean circulatory filling pressure and venous resistance. *Methods*: Demographic data and vasoactive data were collected from the electronic health record and collated with high-fidelity physiologic monitoring data. Mean circulatory filling pressure and venous resistance were calculated using clinically validated equations and then were modeled using a random forest regression incorporating postoperative time and infusion doses of epinephrine, norepinephrine, milrinone, vasopressin, phenylephrine, calcium, sodium nitroprusside, and nicardipine. Similar models were constructed for indexed systemic vascular resistance, cardiac index, cerebral oxygen extraction, and renal oxygen extraction. *Results*: Data from a total of 57 unique patients comprising 9,654,239 data points were analyzed. The model explained 57% of the variance in mean circulatory filling pressure and 59% of the variance in venous resistance. Vasopressin and norepinephrine were the most influential for mean circulatory filling pressure and venous resistance. *Conclusions*: Vasoactive medications appear to modulate venous tone and impact mean circulatory filling pressure and venous resistance. High-fidelity physiologic data allow for characterizing these effects and guide titration of vasoactive medications at the bedside.

## 1. Introduction

Clinical management in the pediatric cardiac intensive care unit is often directed toward maintaining arterial pressure and cardiac output. Although oximetric indices, such as venous oxyhemoglobin saturation, are inherently important physiologic parameters, they are non-specific markers of the adequacy of the circulation; they may effectively indicate inadequate oxygen delivery but are ineffective in distinguishing its cause. They inform *that* something should be done, but not *what* should be done [1,2,3,4].

Arthur Guyton’s model of circulatory physiology emphasized the critical importance of venous return in determining cardiac performance. In this framework, the mean circulatory filling pressure represents the equilibrium pressure throughout the vasculature when cardiac output ceases and reflects the degree of vascular filling relative to compliance. Venous resistance determines how effectively this stored potential energy drives flow back to the right atrium. Together, mean circulatory filling pressure and venous resistance define the operating point at which venous return intersects cardiac output [5,6,7,8].

The venous system consists of a compliant splanchnic reservoir and a peripheral venous compartment, arranged in parallel and series. The splanchnic circulation serves as a dynamically regulated capacitance reservoir that is recruited through venoconstriction or sequestered through venodilation. Blood can flow from the peripheral venous system, through the splanchnic system, and back to the peripheral system, which reflects its series circulation. Blood can also be circulated within the splanchnic system, using its reservoir functionality, which reflects its parallel circulation. Despite its physiologic importance, the venous circulation receives limited clinical attention, especially in pediatric populations [9]. Quantitative data describing the effects of vasoactive medications on venous tone, mean circulatory filling pressure, and venous resistance are sparse, and no prior studies have described these relationships in the setting of pediatric heart transplant recipients.

The primary aim of this study was to characterize the effects of commonly used vasoactive medications on mean circulatory filling pressure and venous resistance following pediatric heart transplantation. Secondary aims were to assess their effects on systemic vascular resistance, cardiac index, renal oxygen extraction, and cerebral oxygen extraction.

## 2. Methods

### 2.1. Study Design

This retrospective study included pediatric patients younger than 18 years of age who underwent heart transplant at a single institution. Eligible patients had continuous arterial blood pressure monitoring via an arterial catheter and central venous pressure monitoring via a central venous catheter. Patients receiving mechanical circulatory support were excluded.

This study was reviewed and approved by the local institutional review board and conducted in accordance with the Helsinki declaration.

### 2.2. Variables of Interest

High-frequency physiologic data were collected from the Sickbay platform (Medical Informatics Corporation, Houston, TX, USA) for the first 48 postoperative hours. Heart rate was obtained from telemetry; systolic, mean, and diastolic blood pressures were obtained from the arterial catheter; respiratory rate and arterial saturation from pulse oximetry, and central venous pressure from the central venous catheter. Data were recorded at 1 s intervals.

Vasoactive infusion doses were recorded as follows: epinephrine, norepinephrine, milrinone, sodium nitroprusside, and nicardipine in mcg/kg/min, calcium chloride infusion in mg/kg/hr, and vasopressin in milliunits/kg/min. Data for vasoactive infusions were extracted from the medicine administration record in Epic (Epic Systems Corporation, Verona, WI, USA).

Oxygen consumption was estimated using the equation outlined by Seckeler: 242.1 + (9.7 × ln age) − (34 × ln weight) − (9.6 × single ventricle) − (11.2 × critical illness). This equation was chosen for its superior performance across age groups [10].

Cardiac index was calculated by the Fick equation using the estimated oxygen consumption and renal near-infrared spectroscopy value as the surrogate for the venous oxygen saturation.

Indexed systemic vascular resistance was calculated using the following equation: (mean arterial pressure − central venous pressure) × 80/cardiac index.

Mean circulatory filling pressure was calculated using the following equation: (0.96 × central venous pressure) + (0.04 × mean arterial pressure) + (0.2 × cardiac output). Venous resistance was calculated using the following equation: (mean circulatory filling pressure − central venous pressure)/venous return [11].

Cerebral oxygen extraction was calculated using the following equation: (arterial oxygen saturation − cerebral near-infrared spectroscopy)/arterial oxygen saturation. Renal oxygen extraction was calculated using the following equation: (arterial oxygen saturation − renal near-infrared spectroscopy)/arterial oxygen saturation.

Prior to processing, all data were normalized to relative time, defined in seconds, with time 0 corresponding to admission to the cardiac intensive care unit following heart transplant. Missing data were imputed using the patient-specific median values.

### 2.3. Statistical Analyses

Machine learning methods were constructed for each independent variable: mean circulatory filling pressure, venous pressure, indexed systemic vascular resistance, cardiac index, renal oxygen extraction, and cerebral oxygen extraction. Independent predictors included relative time, patient identifier, age, weight, and vasoactive infusion doses. Separate random forest regression models were trained for each dependent variable. Each model consisted of 100 trees, a minimum leaf size of two, and bootstrap sampling. Data were split into training and testing subsets in an 80:20 ratio. Model performance was assessed by R^2^ and mean absolute error. Feature importance rankings and partial-dependence plots were generated to quantify and visualize predictor influence.

All statistical analyses were performed using Python (v3.12) on a local Jupyter environment using scikit-learn.

Random forest modeling was used to characterize nonlinear and potentially interacting relationships between vasoactive medication dosing and derived physiologic parameters. Random forests are ensemble, tree-based methods that construct multiple decision trees from bootstrap samples of the data and aggregate their predictions, allowing robust modeling of complex relationships without prespecified functional forms. This approach is well-suited to physiologic data, where effects may be nonlinear, context-dependent, and influenced by interactions between variables.

## 3. Results

### 3.1. Cohort Characteristics

Fifty-seven patients were included. Ten were under 1 year of age, 11 were between 1 and 5 years of age, five were between 6 and 10 years of age, and 20 were over 10 years of age. Median body surface area was 1.23 m^2^ (interquartile range [IQR] 0.57 to 1.86). Median waitlist time was 3.0 months (IQR 1.0–7.0). Median ischemic time was 252 min (IQR 228–279). Median donor age was 15 years (IQR 3–20), and the median donor-to-recipient weight ratio was 1.2 (IQR 1.0 to 1.4).

### 3.2. Mean Circulatory Filling Pressure

The model explained 57% of the variance in mean circulatory filling pressure (R^2^ = 0.57; mean absolute error, 6.61). Feature importance from greatest to least was time, epinephrine, nicardipine, milrinone, vasopressin, calcium chloride, norepinephrine, and nitroprusside (Table 1). Mean circulatory filling pressure decreased with time, nitroprusside, and nicardipine and increased with epinephrine, norepinephrine, and vasopressin (Table 2).

### 3.3. Venous Resistance

The venous resistance model explained 59% of the variance (R^2^ = 0.59; mean absolute error was 5.26). Feature importance ranked time, nicardipine, epinephrine, calcium chloride, milrinone, vasopressin, nitroprusside, and norepinephrine (Table 1). Venous resistance decreased with milrinone, nicardipine, nitroprusside, and calcium chloride and increased with time and epinephrine, norepinephrine, and vasopressin (Table 2).

### 3.4. Indexed Systemic Vascular Resistance

The model explained 90% of the variance in systemic vascular resistance (R^2^ = 0.90; mean absolute error 15.8). Systemic vascular resistance decreased with time, epinephrine, milrinone, nicardipine, nitroprusside, and calcium chloride, and increased with norepinephrine and vasopressin (Table 2).

### 3.5. Cardiac Index

The model for cardiac index explained 16% of the variance (R^2^ = 0.16; mean absolute error 2.60). Cardiac index increased with time, epinephrine, milrinone, nitroprusside, and calcium chloride (Table 2).

### 3.6. Cerebral and Renal Oxygen Extraction

Models explained 90% (R^2^ = 0.90; mean absolute error was 0.01) and 93% (R^2^ = 0.93; mean absolute error 0.01) of the variance in cerebral and renal oxygen extraction, respectively. Both measures decreased with time, epinephrine, milrinone, nitroprusside, and nicardipine, and increased with norepinephrine and vasopressin (Table 2).

**Figure 1:**
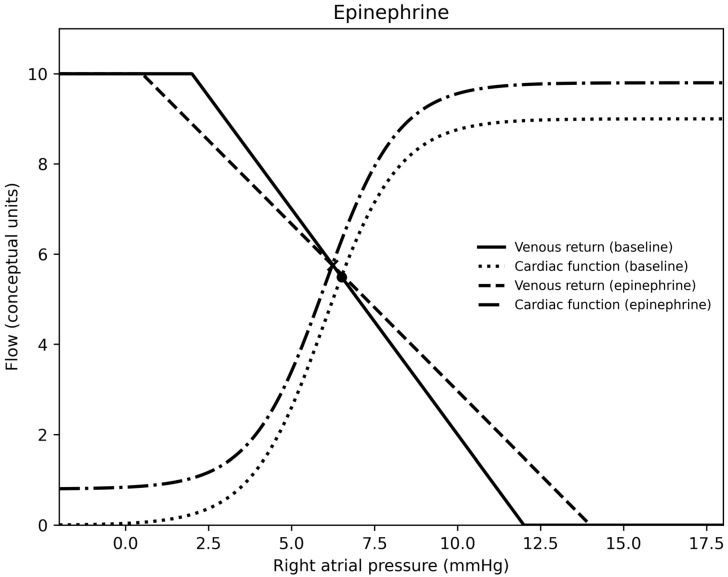
A venous return and right atrial pressure curve depicting the effects of epinephrine.

**Figure 2:**
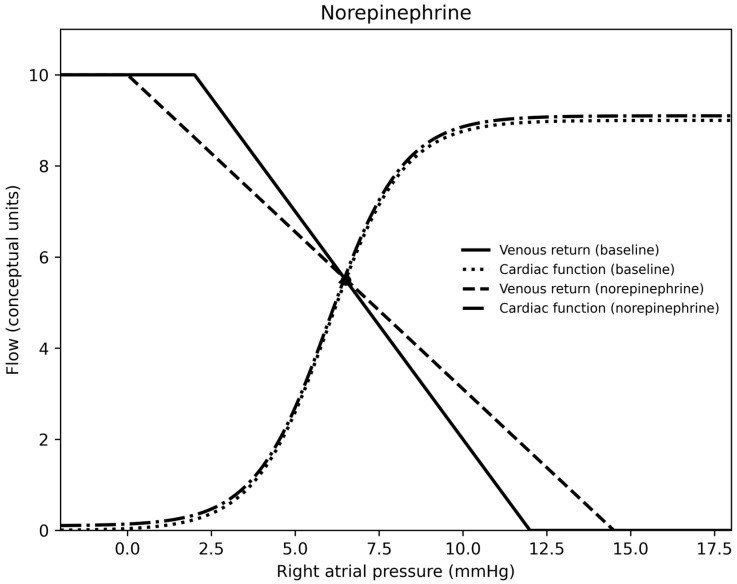
A venous return and right atrial pressure curve depicting the effects of norepinephrine.

**Figure 3:**
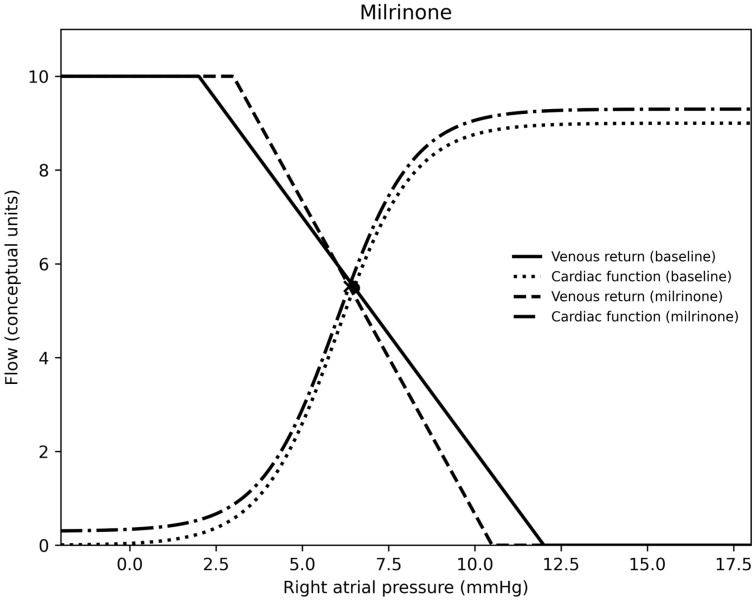
A venous return and right atrial pressure curve depicting the effects of milrinone.

**Figure 4:**
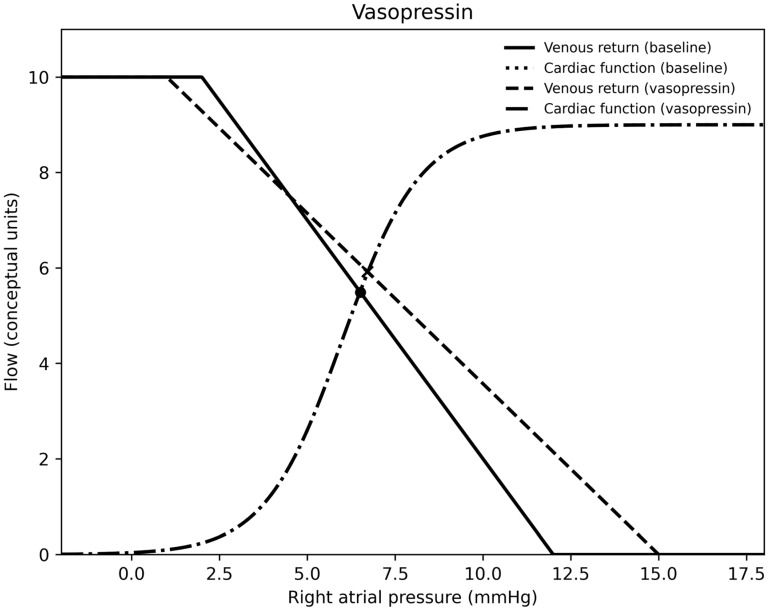
A venous return and right atrial pressure curve depicting the effects of vasopressin.

**Figure 5:**
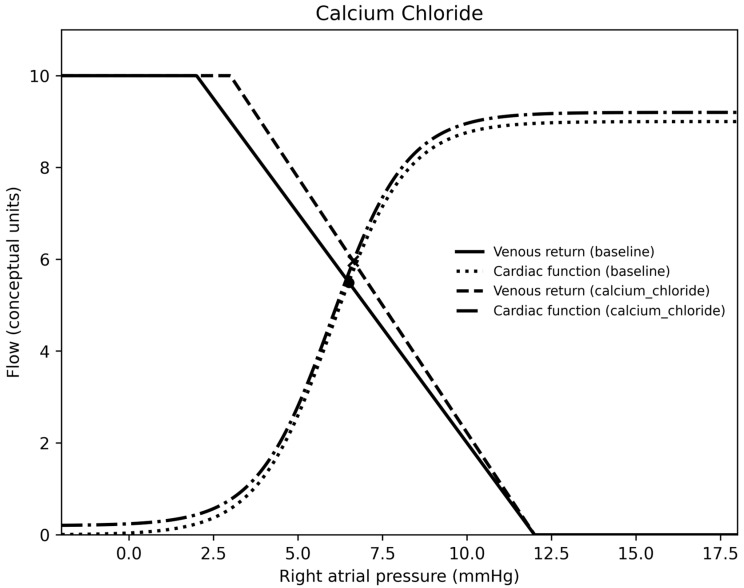
A venous return and right atrial pressure curve depicting the effects of calcium chloride.

In summary, for each medication, epinephrine was a vasodilator (at the doses used) and venoconstrictor, norepinephrine was a vasoconstrictor and venoconstrictor, milrinone was a vasodilator and a venodilator, calcium was essentially neutral on both sides of the circulation, nitroprusside was a vasodilator and venodilator, nicardipine was a vasodilator, and vasopressin was a vasoconstrictor and venodilator. Venous return, cardiac output curves for epinephrine, norepinephrine, milrinone, vasopressin, and calcium chloride are demonstrated in Figure 1, Figure 2, Figure 3, Figure 4 and Figure 5.

## 4. Discussion

High-fidelity physiologic data enable detailed characterization of both arterial and venous circulatory dynamics following pediatric heart transplantation. Approximately 60% of the variance in mean circulatory filling pressure and venous resistance was explained by postoperative time and vasoactive infusions, while systemic vascular resistance and regional oxygen extraction were explained by the model to a much greater extent. In contrast, cardiac index was only weakly predicted by these variables, suggesting that vasoactive infusions primarily modulate vascular tone rather than directly augmenting cardiac output.

Epinephrine was associated with increased mean circulatory filling pressure, venous resistance, and cardiac index, alongside decreased systemic vascular resistance and oxygen extraction. These findings are consistent with venoconstriction and (associated with, if not causing) enhanced contractility. The decrease in systemic vascular resistance occurred from a dose of 0.025 to 0.075 mcg/kg/min, after which systemic vascular resistance increased with increasing epinephrine infusion dose. The rise in mean circulatory filling pressure suggests recruitment of unstressed into stressed volume (i.e., increased preload), and the decrement in systemic vascular resistance likely reflects β2-mediated arterial dilation [12,13,14,15]. The decrease in oxygen extraction suggests enhanced oxygen delivery despite higher venous tone [16]. These findings refine the typical mental model of post-transplant recipients in which arteriolar vasodilation is thought to be paramount to enhanced post-transplant cardiac output, by emphasizing the critical contribution of venous recruitment and preload augmentation. Norepinephrine increased mean circulatory filling pressure, venous resistance, and systemic vascular resistance without a corresponding increase in cardiac index, consistent with concurrent preload and afterload augmentation. Some of this has been demonstrated before [15,17]. The associated rise in oxygen extraction suggests reduced adequacy of oxygen delivery in a high-resistance state.

Milrinone demonstrated venodilation and arterial vasodilation with preserved cardiac index and reduced oxygen extraction, likely reflecting improved ventricular compliance and redistribution of venous blood [18,19,20]. Nitroprusside produced balanced arterial and venous dilation, whereas nicardipine primarily affected arterial tone [21,22,23,24,25]. Calcium chloride appeared largely neutral with modest improvements in cardiac index and oxygen delivery [26].

With high-fidelity physiologic data, there are some special nuances that must be considered. First, the sample size in such a study is the number of data points, not the number of patients, that powers the statistical analyses. Second, “conventional” statistics cannot be practically used for such analyses. While readers may be unfamiliar with the statistical analyses used herein, all are encouraged to use electronic and print resources to familiarize themselves with such methods. As such data becomes more available, such analyses will become more prevalent, particularly since there is ample computing power for such methods in the current era. Machine learning methods such as random forest have already been increasingly employed and are particularly helpful when the dataframe is large, as is the case with the current study, in which there were over 9 million datapoints worth of data. Such physiologic studies can also be prone to mathematical coupling. This phenomenon occurs when the dependent variable is a calculated variable, and the components of it are included as independent variables in the model used to predict it. As the calculated physiologic variables were only used as the dependent variables and not entered as the independent variables in the current study, the risk of mathematical coupling was greatly reduced. The fact that for the same vasoactives there were divergent effects on mean arterial pressure, systemic vascular resistance, and mean circulating filling pressure demonstrates the absence of significant mathematical coupling.

While the current study offers novel insight into the effects of vasoactive medications on the venous and arterial circulations after pediatric heart transplant using high-fidelity physiologic data, it is not without its limitations. First, oxygen consumption relied on an equation that is imperfect. This formula was selected for its overall performance in children of all ages with and without congenital heart disease, but that does not mean it is perfect. Even if the oxygen consumption estimates are not perfect, the hierarchy, direction, and relative strength of associations between variables should be preserved. Consequently, while the exact numerical scale of derived parameters such as cardiac index or systemic vascular resistance may differ depending on the chosen oxygen consumption equation, the overall physiologic relationships and model effects remain valid. Secondly, other variables, such as fluid balance, were purposely not included in the model. The aim of the study was to focus on the vasoactive effect. For the dependent variables except cardiac index, the model performance was quite good. Adding additional independent variables could have helped increase the variability that could be accounted for in cardiac index, but it would not have altered the direction of effect of the vasoactive medications, which was the aim of the study. Additionally, the dataframe was already quite large, and adding additional variables could have significantly increased the required compute and time for model creation. While the statistical power of this study is driven by the number of data points across time, clinical generalizability is still based on the number of patients. Single-center pediatric transplant volume is limited, and thus, this is an issue with any single-center pediatric transplant study. The United States, in its entirety, does approximately 400 transplants a year. Thus, the total of 57 patients is not necessarily a small single-center experience. However, it is important to note that this limits generalizability, and thus this must be taken into account by those reading these data. It is also important to mention that perioperative management followed institutional practices, including general vasoactive medication selection. This is why dopamine and dobutamine were not included in the study, as they are not utilized at our institution. Dosing, titration, and duration of vasoactive support, however, were individualized at the bedside based on real-time physiologic assessment. As a result, substantial variability in medication exposure was present across patients and over time, enabling modeling of medication-associated physiologic effects despite the presence of local practice patterns.

It must be stressed that these findings are all causal and are not causative. These data should be treated as exploratory and as hypothesis-generating.

## 5. Conclusions

In the first 48 h after pediatric heart transplant, vasoactive medications appear to primarily influence vascular tone rather than directly augmenting cardiac output. By shifting mean circulatory pressure and venous resistance, these agents modulate venous return, systemic vascular resistance, and oxygen extraction.

## Figures and Tables

**Table 1:** Feature importance from most important at the top and least important at the bottom for each dependent variable.

Mean Circulatory Filling Pressure	Venous Resistance	Indexed Systemic Vascular Resistance	Cardiac Index	Cerebral Oxygen Extraction	Renal Oxygen Extraction
Time	Time	Time	Time	Time	Norepinephrine
Epinephrine	Nicardipine	Milrinone	Epinephrine	Milrinone	Time
Nicardipine	Epinephrine	Epinephrine	Nicardipine	Epinephrine	Epinephrine
Milrinone	Calcium chloride	Vasopressin	Milrinone	Nicardipine	Milrinone
Vasopressin	Milrinone	Nicardipine	Vasopressin	Vasopressin	Vasopressin
Calcium chloride	Vasopressin	Norepinephrine	Calcium chloride	Calcium chloride	Calcium chloride
Norepinephrine	Nitroprusside	Calcium Chloride	Nitroprusside	Norepinephrine	Nicardipine
Nitroprusside	Norepinephrine	Nitroprusside	Norepinephrine	Nitroprusside	Nitroprusside

**Table 2:** Overview of the direction of effect of vasoactive medications on physiologic variables.

	Mean Circulatory Filling Pressure	Venous Resistance	Indexed Systemic Vascular Resistance	Cardiac Index	Cerebral Oxygen Extraction	Renal Oxygen Extraction
**Epinephrine**	↑	↑	↓	↑	↓	↓
**Norepinephrine**	↑	↑	↑		↑	↑
**Milrinone**	↓	↓	↓	↑	↓	↓
**Calcium chloride**		↓	↓	↑	↓	
**Nitroprusside**	↓	↓	↓	↑	↓	↓
**Nicardipine**	↓		↓		↓	↓
**Vasopressin**	↑	↑	↑		↑	↑

## Data Availability

Data is not publicly available as this is high-fidelity data at 1 s resolution. The dataset is quite large with a high file size and requires a Python workspace to utilize.
